# Evaluating the Impact of the World Health Organization’s Surgical Safety Checklist on Clinical Outcomes and Implementation Strategies: A Systematic Review

**DOI:** 10.7759/cureus.69875

**Published:** 2024-09-21

**Authors:** Lara Alsadoun, Sailakshmn Sanipini, Rafeef Khleif, Abdullah Ashfaq, Abdullah Shehryar, Kaleb A Berhane, Abdur Rehman, Venkata Madusudana Rao Kanukollu, Isa Khan

**Affiliations:** 1 Trauma and Orthopaedics, Chelsea and Westminster Hospital, London, GBR; 2 Medical School, Xavier University School of Medicine, Oranjestad, ABW; 3 Internal Medicine, Xavier University School of Medicine, Oranjestad, ABW; 4 Surgery, Gujranwala Medical College Teaching Hospital, Gujranwala, PAK; 5 Internal Medicine, Allama Iqbal Medical College, Lahore, PAK; 6 Internal Medicine, Adera Medical and Surgical Center, Addis Ababa, ETH; 7 Surgery, Mayo Hospital, Lahore, PAK; 8 Ophthalmology, Stoke Mandeville Hospital, Aylesbury, GBR; 9 Internal Medicine, Nishtar Medical University, Multan, PAK

**Keywords:** clinical outcomes, implementation strategies, surgical safety checklist (ssc), systematic review, world health organization (who)

## Abstract

This systematic review evaluates the impact and implementation strategies of the World Health Organization's Surgical Safety Checklist (WHO SSC) across diverse healthcare settings since its introduction in 2008. Our comprehensive analysis synthesizes findings from various study designs, including randomized controlled trials, qualitative studies, and meta-analyses, focusing on the checklist's effectiveness in reducing surgical complications and enhancing safety cultures within surgical teams. Despite its widespread endorsement and documented benefits, the review highlights significant variability in implementation quality and adherence, influenced by cultural, institutional, and procedural factors. The findings suggest that optimized adaptation and contextual application of the WHO SSC are crucial for maximizing its clinical benefits, particularly in low-resource settings. This review not only confirms the checklist's efficacy in improving surgical outcomes but also underscores the need for evidence-based strategies to enhance its global implementation and effectiveness.

## Introduction and background

The introduction of the World Health Organization's Surgical Safety Checklist (WHO SSC) in 2008 marked a pivotal shift in efforts to enhance patient safety and improve clinical outcomes in surgical environments globally [1]. Rooted in a commitment to reducing surgical errors and preventable complications, the WHO SSC has been extensively adopted and adapted across diverse healthcare settings, encompassing both high-income and low-to-middle-income countries (LMICs) [2]. Empirical research consistently underscores the checklist's potential to foster significant reductions in morbidity and mortality, underscoring its role not merely as a procedural formality but as a catalyst for fostering robust safety cultures within surgical teams [3].

Extensive literature delineates the checklist's efficacy in decreasing rates of surgical complications and enhancing the overall safety of surgical procedures [4]. Studies employing various methodological frameworks, including randomized controlled trials, nonrandomized studies, and qualitative analyses, have contributed to a broad spectrum of evidence supporting the checklist's utility. However, disparities in implementation quality, adherence levels, and contextual adaptations raise compelling questions regarding optimizing the checklist's effectiveness [5]. Moreover, the economic implications of implementing the WHO SSC, particularly in resource-constrained settings, warrant a comprehensive analysis to understand the cost-benefit dynamics in different healthcare systems [6].

Despite the widespread acknowledgment of the checklist's benefits, there exists a heterogeneous landscape of implementation strategies and outcomes, suggesting a complex interplay of institutional, cultural, and procedural factors that influence the efficacy of the WHO SSC [7] [8]. This variability underscores the necessity for a systematic review that not only aggregates outcome data but also critically evaluates the breadth of implementation methodologies and their correlation with clinical outcomes. The primary objective of this systematic review is to synthesize existing research on the implementation strategies and clinical impacts of the WHO SCC across diverse healthcare settings. Specifically, the review aims to elucidate the relationship between various implementation approaches and the resulting clinical outcomes, including morbidity, mortality, and hospital stay lengths. By integrating findings from a wide range of study designs and geographic contexts, this review seeks to identify best practices for checklist implementation that maximize patient safety and operational efficiency, particularly in low-resource settings. Furthermore, this analysis aims to provide evidence-based recommendations for policymakers and healthcare providers to refine surgical safety protocols and improve the overall quality of surgical care globally.

## Review

Materials and methods

Search Strategy

Our search protocol was meticulously aligned with the Preferred Reporting Items for Systematic Reviews and Meta-Analyses (PRISMA) [9] guidelines to gather a comprehensive corpus of literature on the WHO SSC across diverse healthcare settings. We conducted extensive searches across major electronic databases like PubMed, Medline, Embase, the Cochrane Library, and Scopus, covering all records up to August 2024. Keywords and Medical Subject Headings (MeSH) such as "World Health Organization," "Surgical Safety Checklist," and "clinical effectiveness" were strategically combined using Boolean operators to capture a wide spectrum of relevant studies.

Our inclusion criteria focused on peer-reviewed articles, clinical trials, and systematic reviews in English, discussing the clinical impacts and implementation strategies of the WHO SSC. The search also included grey literature like clinical trial registries and conference proceedings to ensure a thorough review of both published and unpublished studies. An expert in healthcare database navigation reviewed our search strategy, ensuring precision and relevance in capturing high-quality evidence on the effectiveness of surgical safety checklists.

Eligibility Criteria

The eligibility criteria for our systematic review were stringently defined to ensure the academic rigor and clinical relevance of the studies focusing on the WHO SSC. Included in the review were peer-reviewed research articles, randomized controlled trials (RCTs), nonrandomized clinical trials, qualitative studies, reviews, and meta-analyses that evaluated the implementation, effectiveness, and clinical outcomes of the WHO SSC in various surgical settings. To be included, studies needed to explicitly examine the implementation strategies or assess the clinical impacts, such as morbidity, mortality, length of hospital stay, and complication rates, associated with the WHO SSC in surgical practices. These studies were required to be in English, covering human subjects within any healthcare setting globally, and providing detailed insights into the checklist's implementation, adaptation, and outcomes, enriching our understanding with historical and contemporary data.

We applied exclusion criteria to omit studies not directly examining the effects of the WHO SSC in surgical environments, including those focusing on general safety interventions without specific reference to the WHO SSC. Excluded were articles that did not report specific outcomes related to the checklist's use or lacked peer-reviewed status, such as conference abstracts, editorial opinions, and unpublished theses. Furthermore, studies with incomplete data or those lacking a clear connection to the checklist's impact on surgical safety and patient care were also excluded to maintain a high standard of evidence and relevance for our systematic review, ensuring a focused and rigorous analysis of the WHO SSC's practical benefits in real-world settings.

Data Extraction

The data extraction process for our systematic review of the WHO SSC was meticulously structured to maximize thoroughness and accuracy. Initially, articles were screened for relevance based on titles and abstracts, with two independent reviewers categorizing them as "relevant," "not relevant," or "possibly relevant." Subsequent full-text reviews of potentially relevant articles were performed using a standardized Microsoft Excel form to ensure uniformity. Each article was assessed against our predefined inclusion and exclusion criteria, with discrepancies resolved through discussion or consultation with a third reviewer. This meticulous process captured essential details such as the lead author, publication year, study design, sample size, key outcomes, and limitations, ensuring comprehensive integration of data crucial for evaluating the effectiveness and implementation strategies of the WHO SSC.

Data Analysis and Synthesis

Given the significant heterogeneity in methodologies and settings of the studies included in our systematic review, we opted for a qualitative synthesis rather than a meta-analysis to explore and integrate the diverse findings related to the WHO SSC. This approach allowed for a nuanced examination of how various healthcare settings adapt to and benefit from the WHO SSC, considering specific operational and cultural contexts. We categorized key findings from each study to identify overarching themes and discernible patterns regarding the checklist's efficacy, barriers to its adoption, and strategies for enhancing its integration into surgical practice. This thematic synthesis provided deep insights into the mechanisms through which the WHO SSC improves surgical safety and highlighted effective implementation practices across different environments, offering a comprehensive overview of WHO SSC utilization and paving the way for future research to optimize its global benefits.

Results

Study Selection Process

**Figure 1:**
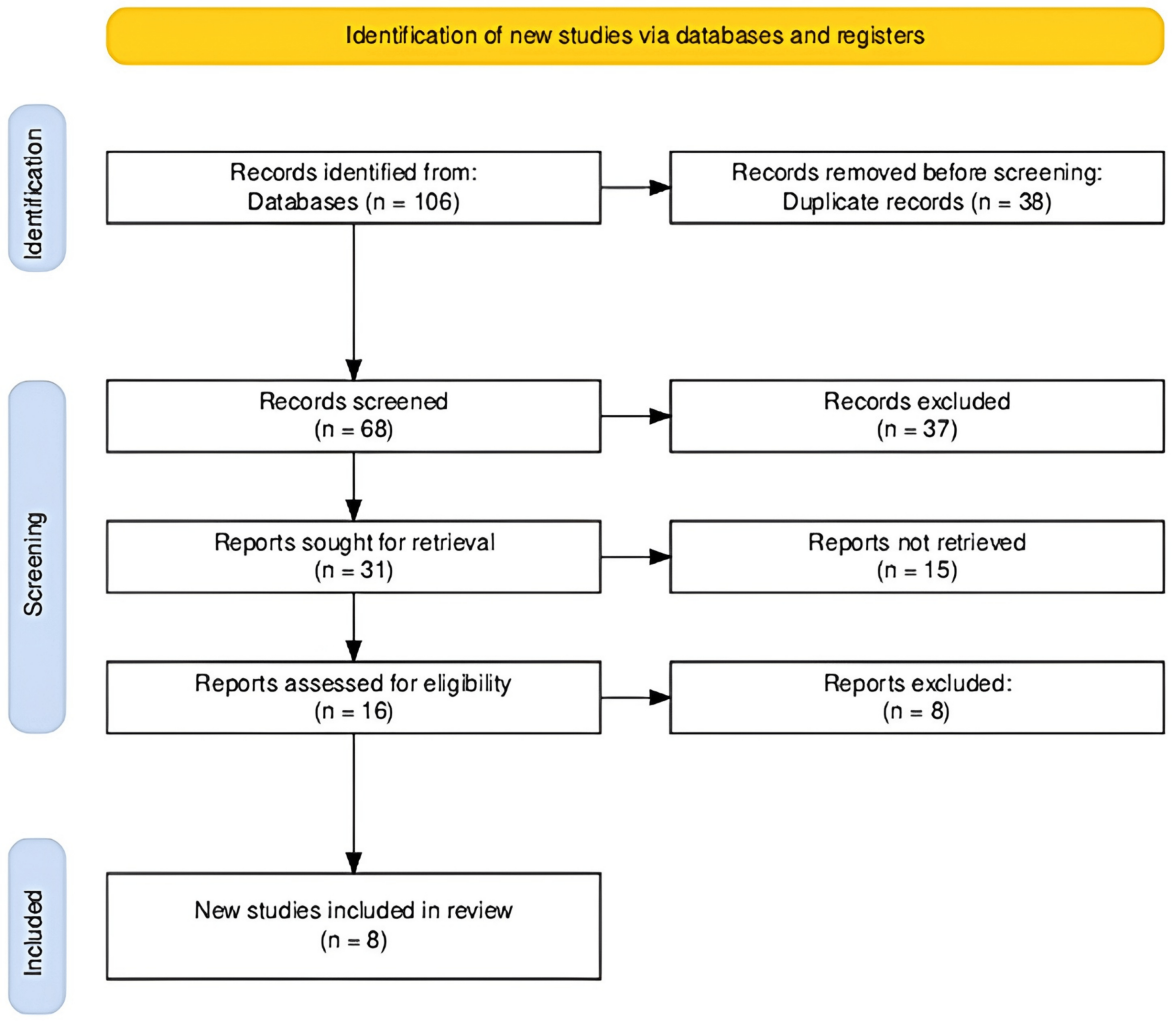
The PRISMA flowchart illustrating the study selection process. PRISMA: Preferred Reporting Items for Systematic Reviews and Meta-Analyses

The study selection process depicted in the flowchart clearly outlines the systematic approach undertaken to identify and select studies for inclusion in the review. Initially, a comprehensive search across various databases yielded a total of 106 records. Following the removal of 38 duplicate records, 68 records were screened based on title and abstract, leading to the exclusion of 37 records that did not meet the predefined relevance criteria. Subsequently, 31 full-text reports were sought for detailed evaluation, out of which 15 could not be retrieved, reducing the pool to 16 reports assessed for eligibility. This assessment phase led to the exclusion of an additional eight reports due to criteria non-compliance or other specified reasons, culminating in the inclusion of eight new studies that met all the necessary conditions for review. This rigorous and structured process ensures the relevance and quality of the studies included, thereby enhancing the reliability and validity of the review findings. The PRISMA flowchart has been provided in Figure 1.

Characteristics of the Selected Studies

The selected studies encompass a range of research designs including qualitative studies, RCTs, nonrandomized trials, reviews, and meta-analyses, each examining the implementation and efficacy of the WHO SSC in various contexts. These studies collectively assess the checklist's impact on surgical safety, cost-effectiveness, patient outcomes, and healthcare processes across multiple countries and healthcare settings. Methodologies varied from extensive prospective trials involving hundreds of patients to systematic reviews of literature and qualitative analyses based on interviews with healthcare workers. Key findings consistently demonstrate reductions in postoperative complications and enhancements in surgical safety culture, although the degree of impact varies, reflecting different implementation strategies and local adaptations. This rich diversity of approaches and outcomes provides a comprehensive view of the WHO SSC's global applicability and effectiveness, with all the detailed information presented in Table 1.

**Table 1:** Key findings and conclusions of the included studies. WHO SSC: World Health Organization Surgical Safety Checklist, SURPASS: Surgical Patient Safety System, LMICs: Low- and Middle-Income Countries, SSC: Surgical Safety Checklist, PROSPERO: International Prospective Register of Systematic Reviews

Authors	Study Type	Objective	Methods	Key Findings	Conclusion
Kristin Harris et al. 2020 [10]	Qualitative Study	Identify risk elements for a patient-driven surgical safety checklist based on the experiences of patients and healthcare workers.	Eleven focus group interviews with 25 post-operative patients and 27 healthcare workers across five surgical specialties in Norway.	Safety risk factors identified under pre-operative and post-operative aspects, including contact information, medication safety, health optimization, preparation details, complication prevention, and follow-up care.	A broad spectrum of risk elements for a patient safety checklist were identified, suggesting that such a checklist could reduce complications and errors in surgical care.
Anette Storesund et al. 2020 [11]	Nonrandomized Clinical Trial	Examine the combined use of SURPASS and WHO SSC on perioperative care outcomes.	Stepped-wedge cluster trial; SURPASS checklists implemented preoperatively and postoperatively in addition to intraoperative WHO SSC across three surgical departments in a Norwegian hospital.	Reduced in-hospital complications, emergency reoperations, and unplanned 30-day readmissions. No changes in mortality or length of hospital stay.	The addition of preoperative and postoperative SURPASS to the intraoperative WHO SSC was beneficial in reducing complications, reoperations, and readmissions, without affecting mortality or hospital stay duration.
Neeraj Chaudhary et al. 2015 [12]	Randomized Controlled Trial	Assess the impact of a modified WHO surgical safety checklist on postoperative outcomes in a tertiary care hospital in a developing country.	Prospective randomized controlled study with 700 patients divided into two groups: one with a modified checklist implementation and a control group.	Significantly lower postoperative complications related to wounds, abdomen, and bleeding. Reductions in overall and higher-grade complications were also observed.	The modified WHO surgical safety checklist significantly reduced mortality and improved various postoperative outcomes, highlighting its effectiveness in a developing country's healthcare setting.
Axel Fudickar et al. 2021 [13]	Review	Review the implementation of the WHO Surgical Safety Checklist and its effects on perioperative morbidity, mortality, and operating-room safety culture.	Review of original publications retrieved by a selective search in PubMed and Medline databases on the Surgical Safety Checklist, analyzing papers published before February 2012.	Significant improvements in perioperative mortality and morbidity across studies. Improved interdisciplinary communication was also noted.	The WHO Surgical Safety Checklist significantly enhances perioperative safety, improves communication and teamwork, and should be implemented as a tool for enhancing safety culture in the operating room.
Andy Healey et al. 2022 [14]	Randomized Controlled Trial	Evaluate the cost-effectiveness of the WHO Surgical Safety Checklist.	Economic evaluation using data from a randomized controlled trial involving 3702 procedures. Costs included checklist implementation, hospital stay, and clinical operating room expenses.	Checklist implementation resulted in additional complication-free admissions, saved bed-days, and reduced costs significantly.	Implementing the WHO Surgical Safety Checklist is a cost-effective measure that enhances surgical safety and reduces hospital costs.
Arvid Steinar Haugen et al. 2019 [6]	Randomized Controlled Trial	Analyze how high-quality implementation of the WHO SSC influences care processes and patient outcomes.	Analysis of 3702 procedures from a stepped wedge cluster randomized controlled trial in Norway, focusing on care process metrics and the quality of SSC implementation.	High-quality SSC implementation was associated with improved care processes such as increased use of warming blankets and appropriate antibiotic administration. Significant reduction in surgical infections and costs associated with blood transfusions.	Effective implementation of the SSC enhances care processes in the operating room, leading to significant improvements in patient outcomes and cost savings.
Michelle C White et al. 2021 [15]	Meta-Analysis	Identify implementation strategies of the WHO SSC in LMICs, evaluate the effectiveness of these strategies, and assess the clinical impact of the SSC.	Systematic review and meta-analysis of studies from Cochrane, MEDLINE, EMBASE, and PsycINFO on SSC use in LMICs. Registered on PROSPERO.	No direct association between implementation strategies and outcomes; however, SSC use significantly reduced mortality and complications, particularly infectious ones, in LMICs.	SSC implementation in LMICs, although varied in strategies, is generally effective, showing high fidelity and significant clinical improvements. More targeted implementation science is needed to optimize outcomes.
Arvid Steinar Haugen et al. 2015 [16]	Randomized Controlled Trial	Evaluate the impact of the WHO SSC on in-hospital morbidity, mortality, and length of stay.	Stepped wedge cluster randomized trial across five surgical clusters at two hospitals, comparing control procedures with procedures using the SSC.	Significant reduction in complication rates and mean length of hospital stay. Mortality decreased significantly in one hospital but not across all hospitals.	The WHO Surgical Safety Checklist significantly reduced complications and length of hospital stay, with some evidence of reduced mortality, demonstrating its efficacy in improving patient outcomes.

Discussion

The systematic review of the WHO SSC reveals a consistent theme across varied healthcare settings: the checklist significantly enhances surgical safety and reduces postoperative complications [17]. This aligns with previous reviews, such as the findings from the seminal article by Haynes et al. [18], which reported significant reductions in both morbidity and mortality following the implementation of the WHO SSC. Similar to the studies conducted by Anette Storesund et al. [11] and Arvid Steinar Haugen et al. [16], these foundational works establish the checklist's effectiveness in reducing in-hospital complications and mortality across different geographical settings.

However, discrepancies arise when examining the depth of impact and the aspects of surgical care affected by the checklist implementation. For instance, Neeraj Chaudhary et al. [12] and Andy Healey et al. [14] observed significant economic benefits and reductions in specific types of complications, such as wound infections and bleeding, which are not uniformly reported across all studies. These variations could stem from differences in the implementation intensity, adherence to the checklist protocols, and the surgical team's engagement with the checklist process. Geographical differences also influence outcomes, as seen in the studies from LMICs by Michelle C. White et al. [15], suggesting that local adaptation of the checklist to meet specific regional needs could play a crucial role in its efficacy. Furthermore, the qualitative study by Kristin Harris et al. [10] emphasizes the importance of integrating patient and healthcare worker experiences in adapting the checklist, a factor not consistently accounted for in quantitative analyses.

The methodological rigor and design of the studies reviewed also contribute to the observed discrepancies. RCTs, such as those conducted by Arvid Steinar Haugen et al. [16], provide strong evidence of causality but might not fully capture the real-world complexities and resistance encountered during the checklist's implementation. By contrast, qualitative studies and nonrandomized trials offer deeper insights into the practical challenges and facilitators of checklist adoption, which are crucial for understanding the variability in outcomes observed across different settings.

Moreover, the implementation context significantly affects the outcomes, as evidenced by the enhanced results seen in settings where comprehensive training and leadership support are emphasized, as discussed by Axel Fudickar et al. [13]. This factor highlights the importance of considering organizational culture and team dynamics when deploying the WHO SSC. The need for tailored approaches to checklist implementation that consider specific institutional and cultural contexts is evident, underscoring the role of customization in achieving optimal outcomes [19]. This review, therefore, not only corroborates the established benefits of the WHO SSC but also advances the discussion on how to overcome barriers to its universal adoption and effectiveness, paving the way for a more nuanced understanding and application of this critical safety tool in surgery.

The practical applications of our findings underscore the transformative potential of the WHO SSC in enhancing surgical safety across diverse clinical settings [20]. The evidence suggests that the checklist's effectiveness can be significantly augmented through contextual adaptations that consider specific surgical environments and cultural norms. For instance, integrating feedback from both healthcare professionals and patients, as highlighted in studies such as by Kristin Harris et al. [10], can tailor the checklist to address unique risks and procedural nuances characteristic of different specialties or local practices. This adaptive approach not only enhances compliance but also improves the overall quality of care by ensuring that all safety concerns are proactively managed [20]. In addition, continuous training and leadership engagement, as shown in the findings from Arvid Steinar Haugen et al. [16], are crucial for maintaining high standards of checklist implementation. By fostering an inclusive and supportive environment, healthcare institutions can maximize the checklist's impact, thereby reducing complications and improving patient outcomes. These adjustments and commitments at the organizational level are essential for the WHO SSC to realize its full potential as a global standard for surgical safety.

The practical implications of this systematic review emphasize the critical role of the WHO SSC in enhancing surgical outcomes across diverse settings [21]. To maximize its effectiveness, the checklist should be adapted to reflect the specific needs and practices of different surgical environments. For example, integrating customized checkpoints that address common complications or procedural nuances pertinent to specific types of surgeries, such as orthopedic or cardiovascular, can enhance the relevance and utility of the checklist in these contexts. In addition, the training of surgical teams on the importance of each checklist item, coupled with regular audits to ensure compliance and identify areas for improvement, can significantly elevate the impact of the WHO SSC [22]. Therefore, by tailoring the checklist to local contexts and maintaining rigorous adherence and review protocols, healthcare providers can optimize surgical safety and patient care outcomes universally.

The methodological rigor of this systematic review is anchored in a meticulously crafted search strategy across multiple databases, ensuring comprehensive coverage of relevant literature, and a robust data extraction process, facilitated by independent reviewers using standardized forms to minimize errors and bias. Analytical methods were carefully chosen to suit the heterogeneity of the included studies, allowing for a nuanced synthesis of both qualitative and quantitative data. However, the review is not without limitations; the diversity in study designs and settings introduces variability that could affect the generalizability of the findings. In addition, the predominance of studies from high-resource settings might limit applicability in low-resource environments, and the exclusion of non-English studies may omit valuable insights. These factors necessitate a cautious interpretation of the results, especially when extrapolating recommendations for global implementation of the WHO SSC.

The systematic review has highlighted several gaps and avenues for future research regarding the WHO SSC. There is a notable deficiency in studies exploring the long-term impacts of the checklist on surgical outcomes and patient safety, particularly in low-resource settings and among diverse populations that may face unique surgical risks or cultural challenges [23]. Further research is needed to assess the sustained effects of the WHO SSC over time, including its integration into routine practice and its influence on patient outcomes beyond the immediate postoperative period [24]. In addition, the potential for integrating the WHO SSC with emerging digital health technologies, such as electronic health records and real-time data feedback systems, presents a promising area for innovation [25]. These technologies could enhance the real-time applicability and monitoring of the checklist, ensuring higher compliance and dynamic updates tailored to individual patient needs and specific surgical contexts. Investigating these aspects will not only address current gaps but also propel the checklist's utility into the evolving landscape of surgical care and patient safety [26].

The findings from this systematic review offer valuable insights for medical education and emphasize the importance of interdisciplinary collaboration in surgical safety. Integrating the WHO SSC into medical training programs can enhance practitioners' understanding and effective use of this tool, ensuring it becomes a fundamental part of surgical practice from the outset of a healthcare professional's career [27]. Training should not only focus on how to use the checklist but also on why each step is crucial, emphasizing the evidence supporting its impact on reducing surgical risks and improving outcomes. Furthermore, the successful implementation of the WHO SSC relies on the collaborative efforts of a diverse team, including surgeons, nurses, anesthesiologists, and support staff, highlighting the necessity of interdisciplinary training sessions that foster a culture of teamwork and mutual respect [28]. By incorporating these elements into medical education, healthcare providers can better appreciate the checklist's role in enhancing patient safety and be equipped to contribute effectively to its implementation and ongoing refinement in their respective roles.

## Conclusions

The systematic review of the WHO SSC underscores its critical role in enhancing surgical safety and improving patient outcomes across diverse healthcare settings. Our findings reiterate that when implemented effectively, the WHO SSC significantly reduces surgical complications and mortality rates, demonstrating its universal value as a tool for surgical teams worldwide. Moreover, the adaptability of the checklist to various clinical environments and cultural contexts highlights its potential as a global standard in surgical care. While there are challenges in implementation, particularly in low-resource settings and among diverse populations, the overarching benefits of the WHO SSC in promoting a culture of safety and teamwork in surgery are clear. As healthcare continues to evolve, the integration of the WHO SSC with new technologies and its continued adaptation to meet the changing needs of surgical teams will be essential. Ultimately, the sustained focus on optimizing and universally applying the WHO SSC will be pivotal in advancing global health outcomes and setting new benchmarks in patient safety and care.
